# Longevity-Promoting Pathways and Transcription Factors Respond to and Control Extracellular Matrix Dynamics During Aging and Disease

**DOI:** 10.3389/fragi.2022.935220

**Published:** 2022-07-07

**Authors:** Tinka Vidović, Collin Y. Ewald

**Affiliations:** ^1^ Tinka Therapeutics, Vrgorac, Croatia; ^2^ Laboratory of Extracellular Matrix Regeneration, Institute of Translational Medicine, Department of Health Sciences and Technology, ETH Zürich, Zürich, Switzerland

**Keywords:** extracellular matrix, healthy aging, matrisome, collagen, mTOR signaling pathway, FOXO transcription factors, NF-κB transcription factor, Nrf2 transcription factor

## Abstract

Aging is one of the largest risk factors for cancer, type 2 diabetes, osteoarthritis, cardiovascular diseases, and other age-related pathologies. Here, we give a detailed description of the interplay of chronic age-related pathologies with the remodeling of the extracellular matrix during disease development and progression. Longevity-promoting signaling pathways slow or prevent age-related diseases. In particular, we focus on the mTOR signaling pathway, sirtuins, and canonical longevity-promoting transcription factors, such as FOXO, NF-κB, and Nrf2. We extend our analysis using chromatin immunoprecipitation (ChIP) sequencing and transcriptomic data and report that many established and emerging longevity-promoting transcription factors, such as CREB1, FOXO1,3, GATA1,2,3,4, HIF1A, JUN, KLF4, MYC, NFE2L2/Nrf2, RELA/NF-κB, REST, STAT3,5A, and TP53/p53, directly regulate many extracellular matrix genes and remodelers. We propose that modulation of these pathways increases lifespan and protects from age-related diseases in part due to their effects on extracellular matrix remodeling. Therefore, to successfully treat age-related diseases, it is necessary to better understand the connection between extracellular matrix components and longevity pathways.

## Introduction

Aging is defined as a time-dependent functional decline of physiological processes. The aging process is one of the highest and most critical risk factors for cancer, type 2 diabetes, cardiovascular diseases, neurodegenerative, and other age-related pathologies. Although the aging process seems to be a stochastic decay of physiological processes and complicated (Freund, 2019; Vertti-Quintero et al., 2021; Statzer et al., 2022b), recently, the field has defined nine hallmarks of aging: genomic instability, epigenetic changes, loss of proteostasis, dysregulated nutrient sensing, mitochondria dysfunction, cellular senescence, stem cell exhaustion, altered intercellular communication, and telomere attrition (Lopez-Otin et al., 2013). One aspect not included in the hallmarks of aging is the recent emerging, yet not understood role of the extracellular matrix during aging and longevity. Here in this review, we focus on extracellular matrix (ECM) remodeling upon longevity-promoting pathway activation. We will give a detailed description of how some of the essential aging signaling pathways and transcription factors influence the extracellular composition and propose that modulation of these pathways increases lifespan and protects from age-related diseases due to their effect on ECM remodeling.

The cells are surrounded by extracellular matrices (ECMs), which are crucial for tissue structure, function, and intercellular communication. The ECM is attached *via* cell surface receptors such as integrins and discoidin domain receptors to the cells. There is a decline in ECM integrity during aging due to the accumulation of damage from collagen fragmentation, oxidation, glycation, cross-linking, and formation of protein aggregates. This decline in ECM integrity leads to loss of organ support and functions and can drive cellular aging and disease progression (Ewald, 2020).

It was shown that cells in a proper environment can live much longer than the maximal lifespan of the animal (Harrison, 1979; Magrassi et al., 2013) and that placing senescent cells into a young ECM rejuvenates them (Choi et al., 2011). Systems-level approaches using mice showed an association with age on the mRNA and protein level (Williams et al., 2022), suggesting dynamic ECM remodeling even during older ages. One of the strongest correlating gene expression signatures with extended lifespan across tissues was *Coro7*, which is important for cytoskeleton remodeling that usually occurs in response to changes in the ECM (Vitiello et al., 2021). Furthermore, overexpressing collagens is required and sufficient for longevity in *C. elegans* (Ewald et al., 2015), indicating an important role of ECM homeostasis in healthy aging. Thus, directly altering ECM composition can slow the aging of the entire organism.

The functions and phenotypes of collagens and other extracellular matrix genes are well conserved, allowing the interchangeability of findings across species for discoveries (Statzer and Ewald, 2020). To enable a complete picture of all proteins that form, remodel, and are associated with the ECM, the matrisome was *in silico* defined and validated with proteomics approaches across several species (Naba et al., 2016; Teuscher et al., 2019a). The extracellular proteins that form the matrix are collagens, proteoglycans, and glycoproteins [ca. 300 proteins; (Naba et al., 2016)]. The remaining ca. 700 proteins are secreted proteases, such as metalloproteases and cathepsins, which remodel the ECM, growth factors, and other proteins associated with the ECM (Naba et al., 2016). In total, the matrisome comprises about 1,000 proteins or 5% of the genome (Naba et al., 2016; Shao et al., 2020). Remarkably, in single-cell RNA sequencing experiments, the matrisome gene expression signatures can predict cell identity and the cellular status during mouse and chick limb development, suggesting that each cell produces its unique ECM (Sacher et al., 2021). Furthermore, the matrisome composition enables cancer-type identification (Socovich and Naba, 2019). This led to the concept of the matreotype, which is a snapshot of the ECM composition associated with or caused by a phenotype or physiological status, such as health or disease (Ewald, 2020). Our group has exploited this concept by using the human GTEx (Statzer et al., 2021) expression data and defining the youthful matreotype to probe against drug gene expression profiles (Statzer et al., 2021). In this way, novel longevity-promoting drugs were identified and validated by lifespan assays using *C. elegans* (Statzer et al., 2021), demonstrating the usability of the matreotype in cross-species approaches. Notably, 41 out of the 47 known longevity-promoting drugs showed significant changes in ECM expression (Statzer et al., 2021), begging the question of what are the underlying molecular pathways covering these health-promoting reprogramming. Thus, we performed a literature search on the reciprocal interactions between longevity-promoting pathways and ECM remodeling. Although many reviews about the key longevity-promoting pathways, such as mTOR, FOXO, NF-κB, NRF2, and sirtuins, exist (Tilstra et al., 2011; Blackwell et al., 2015; Martins et al., 2016; Watroba and Szukiewicz, 2016; Ewald et al., 2018; Weichhart, 2018; Schmidlin et al., 2019), they focus mainly on intercellular changes and extracellular changes are less explored.

## Role of Longevity-Assurance Pathways in ECM Remodeling and Age-Related Diseases

### mTOR Signaling

**FIGURE 1:**
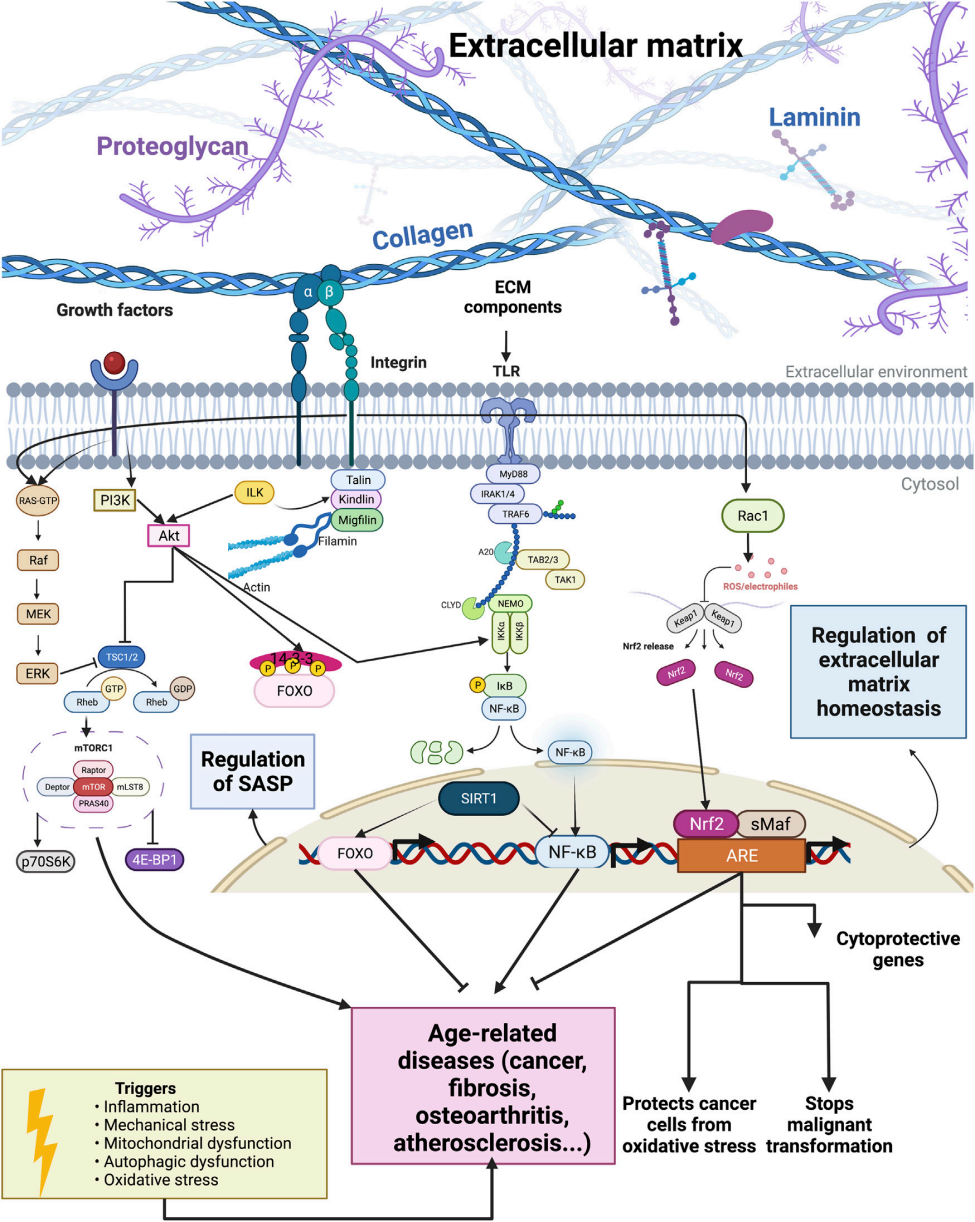
Outside-in and inside-out interplay of longevity pathways and extracellular matrix components. Extracellular matrix components influence longevity pathways and transcription factors. Constitutive activation of the mTOR signaling pathway and transcription factor NF-κB can lead to the development of age-related diseases. By contrast, FOXO, NRF2, and sirtuins activation relate to an increased lifespan.

An important aging pathway that significantly influences ECM remodeling is the mammalian target of the rapamycin (mTOR)/Akt signaling pathway (Figure 1). The mTOR kinase is an evolutionarily conserved protein present in two functionally and structurally different multiprotein complexes, mTORC1 and mTORC2 (Guertin and Sabatini, 2007). The mTORC1 has five subunits: catalytic subunit mTOR, the regulatory-associated protein of mTOR (Raptor), mLST8 subunit, PRAS40, and DEPTOR subunit (Figure 1). PRAS40 and DEPTOR are the negative regulators of the mTORC1 complex (Peterson et al., 2009). The mTORC2 has six subunits: mTOR, rapamycin-insensitive companion of mTOR (Rictor), mSIN1, Protor-1, mLST8, and DEPTOR, where DEPTOR is the only endogenous inhibitor of mTORC2 (Laplante and Sabatini, 2009). The tuberous sclerosis complex (TSC) with two subunits (TSC1 or hamartin and TSC2 or tuberin) regulates mTORC1 activity. The TSC1/2 is a GTPase-activating protein (GAP) for a small GTPase Rheb, and TSC1/2 activation leads to the inactivation of mTORC1 since active GTP-bound Rheb is necessary for mTORC1 activity (Figure 1) (Tee et al., 2003).

The mTORC2 influences cell survival, metabolism, and proliferation *via* phosphorylation and, thus, activation of Akt (Manning and Cantley, 2007). Akt can phosphorylate and therefore inactivate the transcription factor FOXO, which is associated with oxidative stress resistance (Calnan and Brunet, 2008). Furthermore, the mTOR is an essential central controller of ribosome biogenesis, protein synthesis, and cell growth (Jacinto and Hall, 2003). Phosphorylation by mTOR activates p70 ribosomal S6 kinase (p70S6K) and inactivates eIF4E-binding protein (4E-BP1) (Figure 1). Thereby, mTOR kinase influences the translation by preventing the binding of 4E-BP1 to eIF4E, and eIF4E can promote cap-dependent translation. The mTOR also phosphorylates and thereby inactivates retinoblastoma protein (pRb), leading to positive regulation of RNA polymerase I and III activity (Asnaghi et al., 2004).

Phosphorylation of TSC2 by Akt, Erk, or ribosomal S6 kinase inhibits the tumor suppressor complex TSC1–TSC2 (Figure 1). Because the TSC1–TSC2 complex is a negative regulator of mTORC1, its inhibition leads to mTORC1 activation. Activated mTORC1 promotes cell growth and proliferation by promoting translation, and many growth factors act *via* mTORC1 to influence proliferation (Ma and Blenis, 2009). Activated mTORC1 signaling inhibits the insulin–PI3K–AKT pathway, and this negative feedback loop has an essential role in insulin-resistant diabetes and cancer (Easton et al., 2006; Um et al., 2006; Ma and Blenis, 2009). By contrast, reducing mTOR signaling promotes autophagy, mRNA splicing fidelity, hydrogens sulfide signaling, and other mechanisms promoting lifespan increase across species (Harrison et al., 2009; Blackwell et al., 2019; Liu and Sabatini, 2020; Statzer et al., 2022a).

Mechanical stretching can activate the Akt/mTOR pathway *via* β1 integrin (Figure 1), leading to phosphorylation of 4E-BP and ribosomal S6 and causing an increase in the synthesis of collagen, a major structural component of ECM (Mousavizadeh et al., 2020). The composition of ECM exhibits regional variation and dynamic changes related to age, disease, or injury. These changes in ECM can even impact the function of astrocytes. Astrocytes are the most abundant cell type in the central nervous system (CNS), and astrocytes become activated in response to CNS injury, inflammation, or disease. ECM can influence whether astrocytes be in a resting or activated state, and astrocyte wound recovery after mechanical injury varies on different ECM substrates. Johnson et al. tested the recovery of astrocytes grown on different ECM proteins after mechanical stimuli (*i.e.,* scratch) and found that recovery of the scratch was ECM-dependent and that blockage of β1 integrins inhibited astrocyte growth. Therefore, β1 integrin mediates ECM-dependent wound response. They also showed that rapamycin can block wound response on astrocytes grown on tenascin-C and that this effect is mediated by mTORC1 Johnson et al. (2015).

Tissue damage resulting from trauma, disease, or aging can result in organ damage, and tissue regeneration is critical for restoring normal organ functions. Activation of mTOR stimulates the regeneration of neurons and astrocytes, muscles, liver, and intestines, and these effects are mainly mediated by mTORC1. Therefore, disruption of mTORC1 signaling is harmful to tissue regeneration (Wei et al., 2019). Moreover, it was shown that treatment with mTOR inhibitor rapamycin impairs wound healing (Ekici et al., 2007). The mTOR is the master regulator of the inflammatory response in both immune and non-immune cells. Since mTOR plays a critical role in chronic inflammation, it is not surprising that mTOR activation is implicated in many chronic inflammatory diseases (Suto and Karonitsch, 2020). In general, inflammation proceeds to fibrosis (Wick et al., 2010), an excess deposition of amorph collagens (Teuscher et al., 2019b). Thus, inhibiting mTOR can alleviate the chronic inflammatory response, a major driver of fibrotic collagen deposition (Figure 1).

Since tissue regeneration is necessary for restoring tissue homeostasis, mTOR activation in damaged tissue can lead to quicker remission by enhancing tissue regeneration and replacing damaged and lost tissue structures. Therefore, as described in a review from Wei et al. (2019), it is necessary to develop tissue-specific drugs; developing mTOR tissue-specific agonists and antagonists is of great clinical importance. In the very early stages of the disease, when damage to organs is not present, mTOR inhibitors could be more useful in preventing inflammatory damage to organs. Although, in stages where healing and tissue regeneration are happening, tissues may benefit more if a tissue-specific mTOR agonist that increases tissue regeneration is administered. However, since there is a connection between impaired wound healing and the development of tissue fibrosis, this mTOR agonist should be taken very shortly and with great caution.

One of the stages of wound healing is the proliferative phase, in which fibroblasts create the ECM. It was shown that epithelial cells proliferate and migrate faster upon upregulation of the PI3K/mTOR signaling pathway, thereby accelerating wound repair (Castilho et al., 2013). But what if mTOR activation becomes constitutive and causes excessive matrix deposition and reduced ECM remodeling? Fibrosis can happen, and injury is one of the leading causes of fibrosis, and mTOR inhibitors show promise in treating fibrosis (Lawrence and Nho, 2018). Woodcock et al. (2019)showed that the mTORC1/4E-BP1 axis plays a significant role in mediating the fibrogenic effects of TGF-β_1_, the most potent pro-fibrotic mediator. Therefore, targeting the mTOR pathway could be a potentially promising antifibrotic strategy. Aging is a risk factor for fibrotic diseases and increases the likelihood of developing fibrosis upon injury (Murtha et al., 2019). mTOR inhibition increases lifespan and delays age-related diseases in flies, *C. elegans*, and mice (Kapahi et al., 2004; Harrison et al., 2009; Blackwell et al., 2019). Since tissue fibrosis is the leading cause of morbidity and mortality and more than 45% of deaths in the United States can be attributed to fibrotic disorders (Wynn, 2004), one of the reasons why mTOR inhibition increases lifespan may be due to preventing fibrotic disorders in tissues. This hypothesis needs to be still experimentally validated. If it proves to be correct, then the connection between mTOR and excessive matrix deposition could become one of the most important factors for lifespan extension and delay of age-related diseases.

### Sirtuin Activity

Sirtuins (silent information regulator 2) are NAD+ (nicotine adenine nucleotide)-dependent histone deacetylases (HDAC). Histone acetyltransferase (HAT) causes acetylation of lysine residues on a histone or other proteins, while histone deacetylases reverse this reaction. Acetyl-ADP-ribose, nicotinamide, and a deacetylated peptide substrate are products of this deacetylation reaction. Nicotinamide (NAM) is an endogenous inhibitor of sirtuin activity *in vivo* and *in vitro*. The human genome encodes seven sirtuins, and they are found in different cellular compartments: Sirt1, Sirt6, and Sirt7 are mainly in the nucleus, Sirt2 is in the cytoplasm, while mitochondrial sirtuins are Sirt3, Sirt4, and Sirt5 (Michan and Sinclair, 2007).

Osteoarthritis is linked to the changes in Sirt1 activity in the cartilage. Sirt1 plays a role in ECM synthesis and promotes cell survival (Dvir-Ginzberg and Steinmeyer, 2013). It was shown that miR-122 induces ECM damage in chondrocytes by inhibiting Sirt1. The miR-122 expression was significantly increased in osteoarthritis cartilage, while Sirt1 expression was significantly decreased. The overexpression of miR-122 increased the expression of ECM catabolic factors (Bai et al., 2020). Moreover, deacetylation of FOXO4 by Sirt1 could activate the Sox9 expression and maintain the ECM stability of cartilage (Ma et al., 2021). Sirt1 also has an anti-inflammatory effect on chondrocytes by deacetylating and inactivating NF-κB. Therefore, Sirt1 could be a therapeutic target for osteoarthritis (Moon et al., 2013).

Advanced age is associated with aortic stiffening, and interventions that increase Sirt1 also lower age-related aortic stiffness. Machin et al. showed that in transgenic mice that overexpressed Sirt1 aortic elastin was maintained during aging, compared to the wild type that showed a decrease in aortic elastin. Moreover, there were an age-related increase in aortic collagen, advanced glycation end products, and calcification in wild-type mice compared to Sirt1 transgenic mice (Machin et al., 2020). Sirt1 plays a significant role in both bleomycin-induced fibrosis and idiopathic pulmonary fibrosis (IPF). Activation or overexpression of Sirt1 attenuates, while Sirt1 knockdown promotes the pro-fibrogenic activity of TGF-β1 in lung fibroblasts. Therefore, activation or overexpression of Sirt1 could be a therapeutic strategy for IPF (Zeng et al., 2017). Resveratrol, a polyphenol compound that can activate Sirt1, decreases ECM deposition in bleomycin-treated mice by regulation of autophagy, and this process is Sirt1-dependent. Resveratrol treatment reduces fibrotic markers fibronectin, collagen I, and collagen IV and increases the levels of autophagy markers Atg5 and LC3BII (Mise et al., 2014). The overexpression of Sirt1 has a protective function in renal fibrosis, and the protective function is mediated by attenuation of TGF-β1-induced ECM expression (Huang et al., 2014). Expression and activity of Sirt1 decreases in the aged kidney. Another mechanism by which Sirt1 could protect from tubulointerstitial damage in the aged kidney is by deacetylating HIF-1α. It was shown that chronic HIF-1α activation promotes fibrosis. During hypoxia, Sirt1 is downregulated, which allows the activation of HIF-1α. Sirt1 inhibitor sirtinol enhances apoptosis and ECM accumulation. Kidneys from aged mice have more apoptosis and higher expression of ECM proteins (Ryu et al., 2019). The overexpression of connexin 43 also improves renal function in diabetic nephropathy by upregulating Sirt1 expression and enhancing Sirt1-dependent deacetylation and inactivation of HIF-1α (Sun et al., 2020).

Sirtuins control the activity of several longevity-promoting transcription factors. The activities of NF-kappaB, Foxo, and Nrf2, are controlled by Sirt1: deacetylation of NF-kappaB causes inactivation, while Foxo deacetylation results in activation (Figure 1) (Michan and Sinclair, 2007). There were conflicting results about the role of deacetylation on Nrf2 transcriptional activity (Kawai et al., 2011; Ding et al., 2016), most probably because of different cell conditions. Sirt1 activation positively affects lifespan extension and is protective against age-related diseases like diabetes type II, cancer, neurodegenerative, and cardiovascular diseases (Donmez and Outeiro, 2013; Donmez and Outeiro, 2013Imai and Guarente, 2014). Caloric restriction (CR) can activate Sirt1 by upregulating AMPK and increasing cellular levels of NAD^+^, and some of the positive effects of CR on lifespan are mediated by Sirt1. It was also shown that pharmacological induction of sirtuin activity could mimic the positive impact of CR without actually applying CR (Watroba and Szukiewicz, 2016). Sirtuins regulate many functions that have a role in aging, like DNA repair, genome stability, inflammatory responses, apoptosis, mitochondrial function, and cell cycle (Watroba and Szukiewicz, 2016). Therefore, sirtuins cooperate with NF-κB, FOXO, and p53 (Ren et al., 2019) and can indirectly affect the lifespan by regulating these transcription factors.

## Longevity Pathways and Transcription Factors Control ECM Remodeling During Disease and Aging

### FOXO Signaling

The Forkhead family of transcription factors comprises of more than 100 members in humans and, as a characteristic, they contain a conserved DNA-binding domain called the forkhead box (Zanella et al., 2010; Genin et al., 2014). While the invertebrate genome has one Forkhead Box O (FOXO) gene, mammals have four FOXO genes: FOXO1, FOXO3, FOXO4, and FOXO6 (Hannenhalli and Kaestner, 2009). FOXO transcription factors influence and control cell cycle arrest, cell death, metabolism, DNA repair, oxidative stress resistance, and differentiation and can act as a tumor suppressor (Figure 1) (Martins et al., 2016; Tia et al., 2018). Several post-translational modifications, such as acetylation/deacetylation, phosphorylation, ubiquitination, arginine, and lysine methylation, modulate FOXO transcription factors in response to stress (Brunet et al., 2004; Calnan et al., 2012). The FOXO subfamily is an essential transcriptional effector of the insulin/insulin-like growth factor signaling pathway and acts as one of the critical regulators of longevity. Insulin and insulin-like growth factor receptors lead to the activation of the PI3K/AKT pathway, and AKT can phosphorylate FOXO leading to FOXO’s translocation from the nucleus to the cytoplasm (Figure 1). The phosphorylation and inactivation of FOXO by Akt lead to the suppression of FOXO-dependent gene expression (Guo et al., 1999; Murphy et al., 2003; Puig et al., 2003).

The essential molecular pathway of insulin signaling is conserved through evolution (Curran and Ruvkun, 2007). In *C. elegans*, loss-of-function mutations in the Insulin/IGF-1 receptor/*daf-2* signaling pathway can more than double the lifespan of an animal (Kenyon et al., 1993). Recently, it was shown that even at the end of life, degradation of *daf-2* can double the lifespan of very geriatric *C. elegans* (Venz et al., 2021). One of the key regulators of this pathway in worms is the transcription factor *daf-16/*FOXO, which is required for the large lifespan extension produced in *C. elegans* by inhibiting insulin/IGF-1 signaling (Lin et al., 1997; Murphy et al., 2003; Ewald et al., 2018). Willcox et al. (2008) first reported that genetic variation in FOXO3A is strongly associated with human longevity. Several later studies confirmed the association of FOXO3 polymorphisms with extreme longevity (Anselmi et al., 2009; Li et al., 2009). FOXO induces transcription of Sestrin3, a highly conserved antioxidant. Sestrins can activate AMPK, and the exact mechanism of this activation is unknown (Budanov and Karin, 2008). Because of AMPK activation, FOXO can inhibit mTORC1 in a TSC2-dependent manner, and inhibition of mTORC1 is one of the possible mechanisms by which FOXO activation extends lifespan (Hay, 2011). FOXO also, directly and indirectly, regulates several stress response genes, DNA repair genes, proteostasis, metabolism, cell cycle genes, immune function, Parkinson’s disease, and collagen remodeling, thereby improving homeostasis and promoting longevity (Webb et al., 2016).

Fibrosis is a universally age-related disease that involves almost all organs. It is typically initiated by injury and eventually results in excessive collagen deposition, destruction of the organ’s normal structure, and end-organ failure. Forkhead box proteins O1 and O3 (FOXO1/3) have favorable inhibitory effects on fibroblast activation and ECM production and are promising targets for anti-fibrosis therapy. In fibrotic disorders, activated fibroblasts are typically resistant to apoptosis. Therefore, inducing apoptosis of activated fibroblasts may prove effective in fibrotic disorders. FOXO1/3 can modulate the activation and cause apoptosis of fibrogenic effector cells. The fibrogenic effector cells contain fibroblasts and myofibroblasts, active forms of fibroblasts (Xin et al., 2018). Accelerated aging and cellular senescence are driving pulmonary fibrosis. Rehan et al. showed that downregulation of the SIRT3-FOXO3a pathway in idiopathic pulmonary fibrosis (IPF) drives senescent myofibroblast formation and lung fibrosis. Moreover, restoring the SIRT3-FOXO3a pathway leads to apoptosis of senescent myofibroblasts, fibrosis resolution, and lung regeneration. SIRT3/FOXO3a overexpression leads to the upregulation of proapoptotic Bcl-2 proteins. Bcl-2 proteins drive the programmed cell death of senescent myofibroblasts by activating the intrinsic apoptosis pathway (Rehan et al., 2021). Another promising approach is by a peptide (FOXO4-D-Retro-Inverso (DRI)) that blocks the interaction of FOXO4 with p53, which drives senescent cells into apoptosis (Baar et al., 2017). Treating bleomycin-induced pulmonary fibrosis mice with FOXO4-DRI reduced the number of senescent cells and myofibroblasts, attenuated collagen deposition, and downregulated ECM receptors (Han et al., 2022), suggesting a FOXO4-dependent reprogramming to ameliorate pulmonary fibrosis.

Characteristic of cardiac fibrotic diseases (heart failure and diabetic cardiomyopathy) is cardiac myofibroblast (CMF) conversion. TGF-β1 is a crucial protein involved in CMF conversion, and it was shown that FOXO3a is a negative regulator of CMF conversion induced by TGF-β1 (Vivar et al., 2020). Since ECM production and proliferation of fibroblasts also have a role in wound healing, FOXO1/3 activity should not be increased in the early stages of injury to allow injury repair. Still, it should be increased in the late stages of injury to suppress fibrosis and scar tissue formation (Xin et al., 2018).

It is known that vascular smooth muscle cell (VSMC) apoptosis accelerates atherosclerosis and promotes the breakdown of the ECM, and a mechanistic link between these two processes could be FOXO3a. Transcriptional activation of MMP13 (matrix metalloproteinase 13) is mediated by FOXO3a and could explain, at least in part, how FOXO3a activation induces ECM breakdown (Yu et al., 2018). FOXO3a activation not only affects ECM breakdown but can also inhibit fibroblast proliferation. During fibroblast interaction with ECM, there is a proliferation permissive signal that involves activation of the integrin/PI3K/AKT pathway. When fibroblasts are attached to collagen, there is a decrease in PTEN and PP2A phosphatase activity and an increase in PI3K/AKT activity (Figure 1). Both inhibition of PTEN and decrease in PP2A activity inhibit FOXO3a and promote fibroblast proliferation by preventing FOXO3a-mediated upregulation of cell cycle inhibitor p27 (Nho and Kahm, 2010).

Last, osteoarthritis is the most common age-related disease that affects joints, and with aging, the expression of FOXO transcription factors is reduced, which diminishes their chondroprotective effects. Duffy et al. (2020) showed that FOXO transcription factors regulate ECM genes in human chondrocytes. Since increased catabolism in the ECM of the articular cartilage is a crucial factor in the development and progression of osteoarthritis (Rahmati et al., 2017), it is possible that by regulating ECM genes, FOXO transcription factors play a significant role in the development and progression of osteoarthritis.

In summary, FOXO1/3/4 are promising targets for anti-fibrotic therapy. When activated, FOXO1/3 can cause apoptosis of fibrogenic effector cells, but FOXO activity should not be increased in the early stages of injury to allow injury repair. Moreover, FOXO transcription factors have chondroprotective effects, and the positive effect of FOXO activation on osteoarthritis may be mediated by the regulation of ECM genes by FOXO.

### Transcription Factor NF-κB

The transcription factor NF-κB is a pivotal mediator of inflammatory responses. NF-κB is composed of five subunits: NF-κB1 (p50), NF-κB2 (p52), RelA (p65), RelB, and c-Rel, and these subunits are bound to specific κB enhancers as homo- or heterodimers (Gilmore, 1999). The p50 and p52 subunits cannot activate transcription on their own because they lack transactivation subunits. Thus, p50 and p52 homodimers are inhibitory subunits of NF-κB (Kang et al., 1992; Plaksin et al., 1993; Driessler et al., 2004). The NF-κB subunits are usually in the cytoplasm bound to the inhibitory IκB family of proteins. There are two signaling pathways essential in the activation of NF-κB: canonical and non-canonical (or alternative) pathways. The canonical pathway responds to various cytokine receptors and involves phosphorylation and ubiquitin-dependent degradation of inhibitory IκB proteins by a multi-subunit IκB kinase (IKK) complex (Figure 1). The IKK complex has two catalytic subunits, IKKα and IKKβ, and regulatory subunit NEMO or IKKγ. The non-canonical pathway connects with phosphorylation and ubiquitin degradation of NF-κB2 precursor protein, p100, resulting in the generation of p52 and nuclear translocation of p52/RelB (Figure 1). Deregulated NF-κB signaling is the hallmark of inflammation, and a better understanding of NF-κB signaling could lead to better therapy of inflammatory diseases (Liu et al., 2017). Since inflammatory diseases increase with aging, it is not a surprise that the overactivation of NF-κB is one of the transcriptional signatures and drivers of aging (Osorio et al., 2016).

Oxidative, genotoxic, and inflammatory stresses lead to the activation of NF-κB, which regulates the expression of genes involved in cell cycle, cellular senescence, apoptosis, and inflammation (Tilstra et al., 2011). Most age-associated diseases share increased inflammation, and NF-κB signaling is upregulated in atherosclerosis, osteoarthritis, neurodegeneration, osteoporosis, and cardiovascular diseases [reviewed in (Tilstra et al., 2011)]. Inflammaging characterizes the activation of innate immunity that happens because of the immunosenescence of adaptive immunity (Franceschi et al., 2000). Inflammaging is connected with an increase in NF-κB activation and a decline in autophagy and apoptosis. Senescence can cause apoptosis resistance, which relates to a senescence-associated secretory phenotype (SASP; Figure 1). SASP is a condition in which senescent cells secrete many factors associated with inflammation and oncogenesis (Coppe et al., 2010). Inflammatory cytokines secreted from senescent cells stimulate NF-κB signaling. The characteristic of senescent cells is a permanent growth arrest, and normal cells become senescent after exposure to genotoxic stress or after a defined number of divisions. Furthermore, the senescent cells can remodel their microenvironment by altering the expression and organization of key ECM components (Mavrogonatou et al., 2019). The expressions of collagen and elastin have shown differentially expressed patterns in senescent cells, depending on the cell type. At the same time, increased levels of different MMPs, ADAM17, and ADAMTS5, were detected in different models of senescence. By contrast, tissue inhibitors of metalloproteinases (TIMPs) are primarily downregulated in senescence (Levi et al., 2020). An aberrant ECM could drive cellular senescence, as described in Blokland et al. review*.* Remodeling and injury can cause the breakdown of the ECM and the release of ECM fragments that act as damage-associated molecular patterns (DAMPs) and activate pattern recognition receptors (PRRs) on cells of the innate immune system. The activation of PRRs induces NF-κB-mediated pro-inflammatory cytokine release, similar to SASP Blokland et al. (2020). When NF-κB signaling is activated, the expression of MMPs is increased. MMPs are responsible for the degradation of ECM, and these changes might even contribute to skin wrinkling, which is characteristic of premature skin aging (Pittayapruek et al., 2016). Furthermore, the degradation of the ECM is one of the first steps in tumor cell invasion. One of the MMPs, MMP-9, is associated with cancer invasion and metastasis and can lead to the development of several inflammatory diseases, diabetes, cardiovascular, and neurodegenerative diseases (Mondal et al., 2020).

Arterial aging increases the incidence and prevalence of cardiovascular diseases. Some of the characteristics of aged arteries are ECM deposition, elastin fracture, and matrix calcification/glycation (Wang et al., 2014). Inflammation drives the activation of MMPs, which degrade extracellular matrices that can lead to hypertension and atherosclerotic effects within the arterial wall. Hence, MMP inhibition can attenuate age-associated pathophysiologic arterial remodeling in animal models of hypertension and atherosclerosis (Wang et al., 2015).

Shear stress can lead to the activation of NF-κB depending on the ECM composition. For instance, endothelial cells plated onto a collagen or laminin matrix do not activate NF-κB upon shear stress, but when plated onto fibronectin and fibrinogen activate NF-κB in response to shear stress (Orr et al., 2005). Fibronectin and fibrinogen depositions are found in vasculature sites that are prone to develop of atherosclerosis, and thus, Nf-κB might be a driver in the development of atherosclerosis (Kutuk and Basaga, 2003). Furthermore, Li et al. (2013) showed that high pulsatility flow over-polarizes the endothelial cells, leading to NF-κB activation, while normal pulsatile flow that moderately polarizes cells could protect cells. Cellular polarity describes the asymmetric distribution of biomolecules, cellular components, and structures that is transmitted to new cells during cell division. This asymmetry is required for correct cell division, differentiation, motility, and cell migration (Bornens, 2008; Lee and Vasioukhin, 2008; Budovsky et al., 2011). Disruption of epithelial cell polarity can even cause cancer development and affect its progression. Many processes dependent on cell polarity are compromised during aging (e.g., leakier epithelial barriers, older adults have impaired vascular function, and an increased risk of developing cancer). Therefore, it could be that cellular polarity has a significant role in aging and cellular senescence (Soares et al., 2014), and inappropriate ECM composition and NF-κB activation might be underlying drivers of this.

Remodeling the ECM is one of the hallmarks of tumor progression to more advanced stages. ECM remodeling in tumors is characterized by the stiffening of normally wavy collagen bundles into thick linear bundles. Fischer et al. (2021)proposed that wavy ECM bundles are physical barriers for cancer cells and can also serve as a “cell polarization barrier” where high amplitude waves depolarize tumor cells and prevent their directional migration and dissemination, suggesting that the microenvironment of solid tumors has a significant role in tumor progression. It was also shown that the fibronectin matrix is upregulated in lung cancer, and fibronectin is known to contribute to both tumor metastasis and chemotherapy resistance. Cho et al. (2020)showed that lung cancer cells seeded onto the 3D model of tumor-associated ECM activates the TLR4/NF-κB signaling pathway, leading to an increase in IL-8 production. Since IL-8 contributes to the resistance of lung cancer cells *via* EGFR inhibitors (Liu et al., 2015; Fernando et al., 2016), targeting molecular mechanisms that connect an increase in the fibronectin content to an increase in Nf-κB signaling and IL-8 production could be a therapeutic strategy. The cancer cells need to acquire a mesenchymal phenotype to metastasize, which includes enhanced migratory capacity (Kalluri and Weinberg, 2009).

Nf-κB plays one of the major roles in epithelial–mesenchymal transition (EMT), the first step of the metastatic process. Activated NF-κB induces multiple EMT transcription factors, including Twist1, Snail2, and ZEB1/2 (Chua et al., 2007; Storci et al., 2010; Li et al., 2012). Some ECM molecules, like type I collagen, are also implicated in EMT. Type I collagen promotes EMT through integrin-linked kinase (ILK). ILK by inducing phosphorylation of AKT (Figure 1), GSK-3β, and IκB causes activation of the NF-κB, Snail, and LEF-1 transcription factors. An increase in the expression of these transcription factors reduces the expression of epithelial marker E-cadherin. At the same time, it promotes the expression of mesenchymal marker vimentin, which leads to stimulation of EMT and cell migration (Medici and Nawshad, 2010).

In summary, changes in ECM composition upon injury or mechanical stress can lead to NF-κB activation, which is implicated in atherosclerosis and plays a significant role in EMT and the progression of tumors.

### Transcription Factor Nrf2

Nrf2 (nuclear factor E2-related factor 2) is a conserved cellular sensor of chemical- and radiation-induced oxidative stress. It binds to the antioxidant-response element (ARE) and regulates the expression of the ARE-mediated antioxidant genes (Figure 1) (Blackwell et al., 2015). Nrf2 belongs to a subset of basic leucine zipper genes, and a cytosolic inhibitor Kelch-like ECH-associated protein 1 (Keap1) continuously targets Nrf2 for degradation. Oxidative stress causes Nrf2 release from Keap1, translocation to the nucleus, binding to ARE, and activation of gene expression (Figure 1) (Kaspar et al., 2009). Increasing oxidative stress is the characteristic of aging, and it is implicated in the development of age-related diseases. Nrf2 activation is impaired with aging, and induction of many antioxidant genes declines with aging. Changes in the levels of Keap1 during aging might be a reason for the impaired activity of Nrf2 during aging (Zhang et al., 2015). The cells are more sensitive to oxidative stress, endoplasmic reticulum stress, and protein aggregation when NRF2-mediated transcription is decreased, which promotes the aging phenotype. Reduced Nrf2 activity can increase oxidative stress and lead to the development of age-associated diseases (Schmidlin et al., 2019). Therefore, Nrf2 activation could be a promising strategy for preventing age-related diseases.

Oxidative stress has been increasingly recognized to be implicated in the development of osteoarthritis (Bolduc et al., 2019). Kubo et al. (2022)showed that Nrf2 deficiency results in SOX9 suppression and that in older age, Nrf2-KO mice develop an osteoarthritic articular cartilage degeneration. Sox9 has an essential role in regulating cartilage ECM homeostasis, and it can regulate chondrocyte differentiation by promoting type II collagen expression (Akiyama, 2008). Sox9 can also inhibit ECM degradation by inhibiting RUNX2, a key regulator of osteoarthritis development and progression (Orfanidou et al., 2009). Thus, Nrf2, by regulating the expression of SOX9, helps to protect cartilage ECM homeostasis (Kubo et al., 2022).

Oxidative stress is implicated in the development of chronic obstructive pulmonary disease (COPD), and Nrf2 could be a therapeutic target for the intervention and prevention of COPD (Boutten et al., 2011). The transcription factor Nrf2 can also protect against pulmonary fibrosis. Bleomycin is an antineoplastic drug that can cause pulmonary fibrosis. It was shown that bleomycin induces higher protein levels of MMP2, TGF-β, connective tissue growth factor (CTGF), and tenascin-C in *Nrf2* ^*−/−*^ mice than in *Nrf2* ^*+/+*^ mice. Deletion of Nrf2 causes increased susceptibility to pulmonary fibrosis induced by bleomycin, and Nrf2 could protect against fibrogenesis (Cho et al., 2004). Sulforaphane, an Nrf2 activator, has antifibrotic effects on the liver, lungs, and muscles. Sulforaphane can reduce muscle fibrosis in *mdx* mice through Nrf2-mediated suppression of TGF-β/Smad signaling and fibrogenic gene expression (Sun et al., 2016). Astaxanthin, a carotenoid compound with high antioxidant activity, was shown to have a reno-protective effect and can inhibit high glucose-induced renal fibrosis by promoting Nrf2/ARE signaling. Induction of Nrf2 signaling by astaxanthin leads to the inhibition of TGF-β, fibronectin, and ICAM-1 expression induced by high glucose (Xie et al., 2018). A study from Shin et al. suggests that Nrf2/HO-1 signaling plays a protective role against cyclosporin A-induced renal fibrosis by modulating EMT gene changes. EMT plays a crucial role in the metastatic process and can cause fibrosis since generated myofibroblasts are a primary source of ECM production Shin et al. (2010). Nrf2 has a protective effect in streptozotocin-induced diabetic nephropathy by inhibiting TGF-β and reducing ECM production (Jiang et al., 2010).

During aging, skin becomes thin and drastically loses collagen. Lee et al. showed that the skin thickness of *Sod3^+/+^* overexpressing mice was increased during aging compared to wild-type mice. Extracellular superoxide dismutase or SOD3 can promote collagen production in aged mice by activating AMPK and Nrf2/HO-1 pathways. Hence, the extracellular SOD3 and Nrf2 could be potential therapeutic targets for antiaging in the skin (Lee et al., 2021). Transcription factor Nrf2 could also be a potential therapeutic target for cataracts. The incidence of cataracts increases with aging, and some of the flavonoids that can activate Nrf2 have protective effects from cataract formation by increasing Nrf2 activity and attenuating MMP-9 expression (Hilliard et al., 2020).

Nrf2 is considered both a tumor suppressor and oncogene regarding cancer (Sporn and Liby, 2012). Its antioxidant function could mediate both tumor-suppressing and oncogenic effects (Milkovic et al., 2017). Indeed, reactive oxygen species (ROS) participates in all processes in tumorigenesis, from cancer initiation to malignant transformation. ROS can cause oxidative DNA damage and mutation in tumor suppressors and proto-oncogenes. Low levels of ROS are associated with increased cell proliferation and survival (Ivanova et al., 2016). Because of Nrf2’s antioxidant function, it could be that Nrf2 activation by drugs could be used to prevent cancer and other diseases. But what if Nrf2 becomes constitutively activated in cancer cells? High levels of ROS are associated with apoptosis, the release of cytochrome c in the cytosol, oxidation of cytochrome c, and only oxidized cytochrome c can activate caspases (Brown and Borutaite, 2008). High ROS levels could lead to the apoptosis of cancer cells, and the antioxidant function of Nrf2 will protect cancer cells from oxidative stress and apoptosis.

Nrf2 activation can prevent cancer development, but Nrf2 inhibition could lead to cancer cell death if malignant transformation is already present. Similar to cancer, there are conflicting evidence about the role of Nrf2 in senescence. It was long thought that the relationship between Nrf2 and cellular senescence is an inverse one, where reduced Nrf2 activity correlates with increased senescence (Fulop et al., 2018; Yuan et al., 2021). Recently, Hiebert et al. (2018) identified the unexpected role of Nrf2 in the induction of cellular senescence and cancer-associated fibroblast (CAF) phenotype through the regulation of matrisome. Activating Nrf2 in fibroblasts promotes cellular senescence by producing a senescence-promoting matrisome, leading to accelerated wound closure and increased tumor growth (Hiebert et al., 2018; Hiebert, 2021). In summary, it could be that depending on cell type and cellular fitness, Nrf2 activation could lead to both positive and negative effects. It is possible that in young and non-senescent cells, Nrf2 activation inhibits tumorigenesis, but old and senescent cells can lead to increased cancer cell survival. Thus, Nrf2 activity needs to be tightly, time-dependent, and tissue-specifically controlled to enable beneficial and health-promoting effects.

## Matrisome Genes as Targets of Longevity Transcription Factors

### Longevity transcription Factors Can, Directly and Indirectly, Regulate Matrisome Genes

As outlined above, the key canonical longevity pathways are interconnected and play major functions in preventing age-related diseases. Dysregulation of ECM composition is a major driver of many pathologies, such as high quantity and amorph deposition of collagens in fibrosis, increased ECM stiffness during cancer, altered ECM secretion of senescent cells (SASPs), or degradation of ECM in osteoarthritis. Previously, we described that many longevity pathways could influence the dysregulation of ECM remodeling. We discussed that the extracellular matrix components could regulate longevity transcription factors, that is, outside-in signaling (Figure 1). Outside-in signaling usually results in an inside-out signaling since cells respond and adapt to their surrounding ECM (Qin et al., 2004). This ensures bidirectional signaling across the plasma membrane to adapt cell function to its environment. Thus, we hypothesized that these longevity-promoting transcription factors that respond to changes in the ECM (*i.e*., outside-in signaling) would also directly participate in signaling from the inside-out. This implies that these transcription factors would transcribe ECM or matrisome genes that would adapt to the extracellular environment, including remodeling of the ECM.

To address this hypothesis, we used the Harmonizome database, which contains a collection of processed datasets from over 70 major online resources (Rouillard et al., 2016) to find target genes of transcription factors from published chromatin immunoprecipitation sequencing (ChiP-seq) studies. We used “CHEA Transcription Factor Targets” dataset (Lachmann et al., 2010) from the Harmonizome database to obtain transcription factor gene targets. We extended our list of longevity transcription factors and included several other important but less established longevity-promoting transcription factors that we have not covered in detail earlier. These are the STAT and GATA family of transcription factors, AP-1/JUN, HIF1A, MYC, CREB, KLF4, REST, and TP53. Fischer et al. (2022) review in detail all transcription factors that are regulators of longevity. In this way, we obtained a more comprehensive analysis.

**FIGURE 2:**
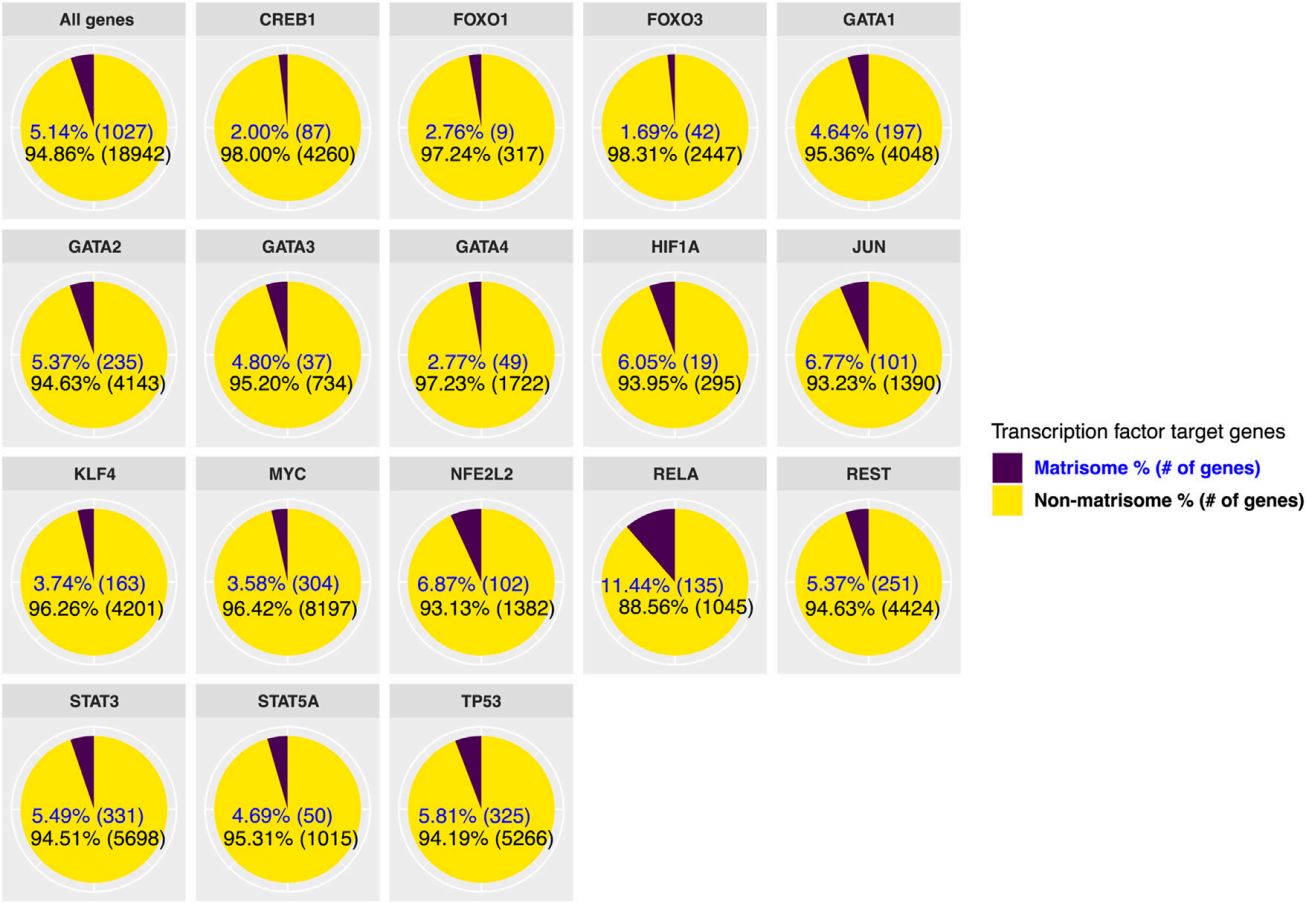
Direct regulation of matrisome genes by longevity transcription factors. We used previously published ChIP-seq studies to find target genes of transcription factors that are associated with longevity. We divided the target genes of each transcription factor into matrisome and non-matrisome genes. From selected longevity transcription factors, MYC regulates the highest number of overall genes, while the largest number of matrisome genes is regulated by STAT3.

Next, we stratified target genes of transcription factors as matrisome and non-matrisome genes (Figure 2; Supplementary Data S1). There are 1,027 matrisome genes or 5% of the genome (Naba et al., 2016; Shao et al., 2020; Statzer and Ewald, 2020), and we observed that the longevity transcription factors directly bind between ∼2–11% of matrisome genes out of the total genes they regulate (ranging from 9–331 matrisome genes compared to 314–8,501 total genes; Figure 2 **)**. Taking the 5.14% of the genome are matrisome genes as the baseline, then 4 out of 17 transcription factors (HIF1A, JUN, NFE2L2/Nrf2, RELA/ NF-κB) assessed here showed significant enrichment for binding onto matrisome genes (>6% matrisome genes; Figure 2). While FOXO1 and FOXO3 only bind 2.8% and 1.7% of matrisome genes, respectively (Figure 2), a previous extensive analysis of conserved FOXO targets revealed that collagen remodeling genes are among the FOXO targets that change with age (Webb et al., 2016), suggesting that these few matrisome genes may be sufficient to repair the underlying tissue deterioration during aging. On the other hand, the second most enrichment for matrisome genes was observed with the Nrf2 ChIP data (Figure 2). In addition to re-establishing redox balance, Nrf2 is crucial for wound healing and recently has been shown to target matrisome genes to drive cellular senescence (Hiebert et al., 2018; Hiebert, 2021). The highest enrichment for matrisome genes was observed with RELA/NF-κB (Figure 2), suggesting that NF-κB not only regulates inflammation but also might directly orchestrate ECM remodeling. Thus, these ChIP-seq data suggest that longevity-promoting transcription factors directly target ECM genes.

To identify common ECM remodelers of longevity transcription factors, we listed collagen, and ECM regulators (*e.g.*, ECM proteases and inhibitors, ECM crosslinkers, *etc*) in Table 1. The most common collagen targets were collagen type IV (11/17 TF), V (8/17 TF), I (7/17 TF), XVIII (6/17 TF), and VI (5/17 TF), and the most common ECM regulators were ADAMTS5, EGLN3, SULF2, P4HA2, PAPPA, FAM20B, and ADAM23 (#/17 TF stands for the number of transcription factors out of the total 17 transcription factors; Table 1). However, in general, there was a poor overlap of ECM remodelers across the different transcription factors, suggesting tissue/disease-specific regulation.

**TABLE 1:** List of transcription factor target genes from ChIP-seq studies stratified to collagen and ECM regulators matrisome gene categories.

	CREB1	FOXO1	FOXO3	GATA1	GATA2	GATA3	GATA4	HIF1A	JUN	KLF4	MYC	MYC	NFE2L2	RELA	REST	STAT3	STAT3	STAT5A	TP53	TP53
Collagen	*COL13A1*		*COL2A1*	*COL12A1*	*COL18A1*	*COL17A1*	*COL12A1*		*COL12A1*	*COL14A1*	*COL13A1*	*COL27A1*	*COL14A1*	*COL1A2*	*COL11A1*	*COL11A1*	*COL26A1*	*COL16A1*	*COL11A2*	*COL4A3*
	*COL15A1*			*COL15A1*	*COL19A1*	*COL4A1*	*COL4A4*		*COL22A1*	*COL18A1*	*COL15A1*	*COL28A1*	*COL1A2*		*COL11A2*	*COL12A1*	*COL27A1*	*COL24A1*	*COL12A1*	*COL4A5*
	*COL16A1*			*COL18A1*	*COL1A1*				*COL4A1*	*COL2A1*	*COL16A1*	*COL4A2*	*COL25A1*		*COL13A1*	*COL13A1*	*COL4A3*		*COL13A1*	*COL4A6*
	*COL17A1*			*COL1A1*	*COL23A1*				*COL4A2*	*COL4A1*	*COL18A1*	*COL5A1*	*COL4A1*		*COL16A1*	*COL14A1*	*COL4A6*		*COL18A1*	*COL5A1*
	*COL1A1*			*COL22A1*	*COL25A1*				*COL6A3*	*COL4A2*	*COL19A1*	*COL5A2*	*COL5A1*		*COL17A1*	*COL16A1*	*COL6A1*		*COL1A1*	*COL5A2*
	*COL5A1*			*COL23A1*	*COL26A1*					*COL4A5*	*COL1A1*	*COL5A3*	*COL8A1*		*COL18A1*	*COL17A1*	*COL6A5*		*COL23A1*	*COL6A3*
	*COL9A3*			*COL24A1*	*COL27A1*					*COL4A6*	*COL20A1*	*COL6A1*			*COL22A1*	*COL21A1*	*COL8A2*		*COL24A1*	*COL8A1*
				*COL27A1*	*COL4A5*					*COL5A1*	*COL24A1*	*COL9A1*			*COL4A2*	*COL22A1*			*COL26A1*	*COL8A2*
				*COL2A1*	*COL5A1*					*COL6A1*	*COL25A1*	*COL9A2*			*COL5A3*	*COL23A1*			*COL27A1*	*COL9A3*
				*COL4A2*	*COL9A1*					*COL6A2*					*COL6A5*	*COL24A1*			*COL2A1*	
				*COL4A3*						*COL8A2*					*COL6A6*				*COL4A1*	
				*COL4A4*											*COL7A1*					
				*COL5A1*											*COL8A1*					
				*COL5A3*											*COL9A2*					
				*COL9A1*																
ECM regulators	*ADAM11*	*CELA2A*	*ADAM17*	*ADAM11*	*ADAM11*	*ADAMTS5*	*ADAMTS1*	*EGLN3*	*ADAM12*	*ADAM17*	*ADAM12*	*MMP2*	*ADAM17*	*ADAM19*	*ADAM11*	*A2M*	*PAPPA*	*ADAMTS5*	*ADAM12*	*MMP28*
	*ADAMTS1*	*F10*	*ADAM19*	*ADAM19*	*ADAM18*	*AMBP*	*ADAMTS15*	*LOXL2*	*ADAM23*	*ADAM19*	*ADAM15*	*MMP21*	*ADAM22*	*AGT*	*ADAM12*	*ADAM10*	*PCSK5*	*ADAMTS6*	*ADAM15*	*OGFOD2*
	*ADAMTS17*	*PLG*	*CTSO*	*ADAMTS14*	*ADAM19*	*CST11*	*ADAMTS5*	*P4HA1*	*ADAM30*	*ADAM23*	*ADAM17*	*MMP23B*	*ADAM23*	*CTSB*	*ADAM2*	*ADAM11*	*PLAT*	*ADAMTSL3*	*ADAM19*	*P4HA1*
	*ADAMTS2*	*SERPINA5*	*HPSE*	*ADAMTS17*	*ADAM23*	*CTSF*	*ADAMTS6*	*P4HA2*	*ADAMTS1*	*ADAMTS18*	*ADAM18*	*MMP24*	*ADAMTS6*	*CTSL*	*ADAM20*	*ADAM12*	*PLOD1*	*CTSC*	*ADAM21*	*PAPPA*
	*ADAMTS20*	*SERPINA9*	*ITIH3*	*ADAMTSL4*	*ADAMTS14*	*MMP19*	*ADAMTS8*	*PLOD2*	*ADAMTS12*	*ADAMTS4*	*ADAM2*	*MMP25*	*ADAMTSL4*	*CTSS*	*ADAM21*	*ADAM18*	*PLOD2*	*CTSH*	*ADAM22*	*PCSK6*
	*ADAMTS3*		*LEPRE1*	*ADAMTSL5*	*ADAMTS3*	*P4HA2*	*CST3*		*ADAMTS18*	*CD109*	*ADAM23*	*MMP28*	*AMBP*	*HPSE*	*ADAM23*	*ADAM22*	*PZP*	*FAM20A*	*ADAM30*	*PLOD2*
	*ADAMTS5*		*MMP15*	*CD109*	*ADAMTS4*	*PLOD1*	*CTSA*		*ADAMTS2*	*CST3*	*ADAM29*	*MMP7*	*CST6*	*ITIH2*	*ADAM29*	*ADAM23*	*SERPINA1*	*LEPREL1*	*ADAM8*	*PRSS12*
	*ASTL*		*MMP17*	*CELA1*	*ADAMTS5*	*SULF2*	*HRG*		*ADAMTS5*	*CSTB*	*ADAM8*	*MMP9*	*CSTB*	*MMP1*	*ADAM32*	*ADAM29*	*SERPINA11*	*PZP*	*ADAMTS13*	*PRSS2*
	*CPN2*		*PAPPA*	*CPN2*	*ADAMTS6*		*HYAL2*		*AGT*	*CSTL1*	*ADAM9*	*NGLY1*	*CTSB*	*MMP3*	*ADAM7*	*ADAM7*	*SERPINA3*	*SERPINA11*	*ADAMTS14*	*SERPINA3*
	*CTSL*		*SERPINA11*	*CTSB*	*ADAMTS9*		*KAZALD1*		*CD109*	*CTSW*	*ADAMTS1*	*OGFOD1*	*CTSC*	*MMP9*	*ADAMDEC1*	*ADAM9*	*SERPINB3*	*SERPINB5*	*ADAMTS16*	*SERPINB1*
	*CTSZ*		*SULF2*	*CTSC*	*ADAMTSL1*		*MMP27*		*F9*	*EGLN2*	*ADAMTS10*	*OGFOD2*	*CTSO*	*PAPPA*	*ADAMTS13*	*ADAMDEC1*	*SERPINB7*	*SERPINB6*	*ADAMTS18*	*SERPINE2*
	*EGLN3*			*CTSD*	*ADAMTSL2*		*P4HTM*		*FAM20B*	*F12*	*ADAMTS13*	*P4HA1*	*EGLN3*	*PI3*	*ADAMTS16*	*ADAMTS10*	*SERPINE1*	*SERPINE1*	*ADAMTS19*	*SERPINF1*
	*HPSE2*			*CTSE*	*ADAMTSL4*		*TGM2*		*HTRA3*	*FAM20B*	*ADAMTS16*	*PLG*	*HABP2*	*PLAU*	*ADAMTS2*	*ADAMTS12*	*SERPINE2*	*TGM1*	*ADAMTS2*	*ST14*
	*HYAL2*			*CTSF*	*ADAMTSL5*		*TIMP3*		*HYAL4*	*HTRA1*	*ADAMTS17*	*PLOD1*	*MMP7*	*SERPINA1*	*ADAMTS20*	*ADAMTS16*	*SERPINF2*		*ADAMTS20*	*SULF2*
	*HYAL3*			*CTSL*	*C17orf58*				*ITIH2*	*HYAL1*	*ADAMTS19*	*PLOD3*	*P4HA1*	*SERPINA3*	*ADAMTS4*	*ADAMTS17*	*SERPING1*		*ADAMTS3*	*TGM1*
	*LOXL3*			*CTSS*	*CD109*				*LEPREL1*	*HYAL2*	*ADAMTS2*	*PRSS12*	*PAPPA*	*SERPINB1*	*ADAMTSL1*	*ADAMTS18*	*SERPINI1*		*ADAMTS5*	*TGM3*
	*MMP14*			*CTSZ*	*CELA1*				*LOX*	*HYAL3*	*ADAMTS20*	*PRSS3*	*SERPINA12*	*SERPINB8*	*BMP1*	*ADAMTS19*	*SULF2*		*ADAMTS6*	*TGM6*
	*MMP9*			*EGLN1*	*CTSB*				*LOXL4*	*LOX*	*ADAMTS4*	*SERPINA1*	*SERPINB9*	*SERPINE1*	*CELA2A*	*ADAMTS20*	*TGM2*		*ADAMTSL1*	*TIMP3*
	*OGFOD2*			*EGLN3*	*CTSS*				*MEP1A*	*MASP2*	*ADAMTS5*	*SERPINA10*	*SERPINE1*	*SERPINE2*	*CST11*	*ADAMTS3*	*TIMP3*		*ADAMTSL4*	*TLL1*
	*P4HA2*			*FAM20B*	*EGLN3*				*MMP14*	*MMP11*	*ADAMTS7*	*SERPINA6*	*SERPINF1*	*SERPINH1*	*CST8*	*ADAMTS4*	*TLL1*		*ASTL*	*TLL2*
	*PAMR1*			*HPSE*	*FAM20B*				*MMP16*	*MMP14*	*ADAMTS8*	*SERPINB11*	*SLPI*	*SLPI*	*CSTL1*	*ADAMTS6*			*CD109*	
	*PLOD3*			*HPSE2*	*HPSE2*				*PAPPA*	*MMP15*	*ADAMTSL1*	*SERPINB6*	*SULF1*	*TGM1*	*CTSF*	*ADAMTS9*			*CELA3A*	
	*SERPINB13*			*HTRA3*	*LEPREL2*				*PLAU*	*MMP16*	*AMBP*	*SERPINB9*	*TGM3*	*TGM2*	*EGLN3*	*ADAMTSL1*			*CPN2*	
	*SULF2*			*KY*	*LOXL4*				*PRSS12*	*MMP17*	*BMP1*	*ST14*		*TLL1*	*F12*	*ADAMTSL4*			*CSTA*	
	*TIMP3*			*LOX*	*MEP1A*				*SERPIND1*	*OGFOD1*	*CPN2*	*SULF2*			*FAM20B*	*ADAMTSL5*			*CTSB*	
				*LPA*	*MMP10*				*SERPINE1*	*P4HA2*	*CST3*	*TGM5*			*KAZALD1*	*CD109*			*CTSD*	
				*MASP1*	*MMP2*				*SPAM1*	*P4HTM*	*CST7*	*TGM6*			*KY*	*CPN2*			*CTSW*	
				*MMP14*	*MMP24*				*TLL1*	*PLOD1*	*CTSA*	*TIMP2*			*LOX*	*CST7*			*FAM20A*	
				*MMP15*	*MMP8*				*TMPRSS15*	*PLOD3*	*CTSD*	*TMPRSS15*			*LOXL4*	*CTSO*			*FAM20B*	
				*MMP27*	*P4HA2*					*SERPINA3*	*CTSL*				*MASP2*	*CTSS*			*FAM20C*	
				*MMP8*	*PCSK5*					*SERPINB5*	*CTSZ*				*MMP16*	*CTSZ*			*HPSE*	
				*OGFOD1*	*PCSK6*					*SERPINB6*	*EGLN2*				*MMP24*	*EGLN3*			*HPSE2*	
				*P4HA1*	*PLAT*					*SERPINE2*	*EGLN3*				*OGFOD2*	*F12*			*HTRA1*	
				*P4HA2*	*PLAU*					*SERPINI1*	*ELANE*				*P4HA1*	*F13A1*			*HYAL1*	
				*PAPPA*	*PLG*					*ST14*	*F13B*				*P4HA2*	*FAM20B*			*HYAL2*	
				*PCSK6*	*SERPINB12*					*SULF2*	*F2*				*P4HTM*	*HPSE2*			*HYAL3*	
				*PLG*	*SERPINB2*						*F7*				*PLAT*	*HTRA1*			*KY*	
				*PRSS12*	*SERPINB6*						*HABP2*				*PLOD3*	*KAZALD1*			*LEPREL1*	
				*SERPINB1*	*SERPIND1*						*HTRA1*				*PZP*	*KNG1*			*LOX*	
				*SERPINB9*	*SERPINE1*						*HYAL1*				*SERPINA10*	*KY*			*LOXL3*	
				*SERPINH1*	*SERPING1*						*HYAL2*				*SERPINA5*	*MEP1A*			*LOXL4*	
				*TGM1*	*SERPINH1*						*ITIH1*				*SERPINA6*	*MMP13*			*MMP14*	
				*TGM2*	*TGM1*						*LEPREL2*				*SERPIND1*	*MMP15*			*MMP2*	
					*TGM2*						*LOX*				*SERPINE3*	*MMP20*				
					*TGM3*						*LOXL1*				*SERPINI2*	*MMP21*				
					*TGM4*						*LOXL3*				*SPAM1*	*MMP23B*				
					*TGM6*						*LOXL4*				*SULF2*	*MMP24*				
					*TIMP3*						*MMP10*				*TGM2*	*MMP25*				
					*TLL1*										*TGM4*	*MMP28*				
					*TLL2*										*TLL1*	*MMP9*				
															*TLL2*	*P4HA3*				
															*TMPRSS15*	*PAMR1*				

With the ChIP-seq, we established the matrisome targets; we next wanted to determine the matrisome genes differentially expressed by these longevity transcription factors. From the Harmonizome database (Rouillard et al., 2016), we used the processed Gene Expression Omnibus dataset “Signatures of Differentially Expressed Genes for Gene Perturbations” (Edgar et al., 2002; Barrett et al., 2013) to find mRNA expression profiles for cells or tissues, following a genetic perturbation (knockdown, knockout, overexpression, and mutation). For comparison, we stratified collagen and ECM regulator genes from the list of differentially expressed genes after transcription factor gene perturbation (Table 2). We noticed that there is a difference between a set of matrisome genes that are differentially regulated after transcription factor perturbation and those matrisome genes that are directly regulated by the transcription factor. We noticed little overlaps between the set of matrisome genes that were differentially regulated after transcription factor perturbation and those matrisome genes that were directly regulated by the transcription factor. This could indicate that longevity transcription factors can, directly and indirectly, regulate matrisome genes. However, further studies comparing ChIP-seq with RNA-seq in the same experimental settings are needed to provide stronger evidence for such a conclusion. Since these transcription factors can regulate ECM genes, it is possible that by regulating matrisome genes, these transcription factors influence longevity.

**TABLE 2:** List of collagen and ECM regulator genes differentially regulated after transcription factor perturbation. KD = knockdown; KO = knockout; OE = overexpression.

Transcription factor	Cell or tissue	Species	Perturbation	Collagen genes (downregulated)	Collagen genes (upregulated)	ECM regulators (downregulated)	ECM regulator genes (upregulated)	GEO accession
CREB	K562 myeloid leukemia cell line	Human	Depletion	*COL19A1*	*COL15A1*	*ADAM23*	*PRSS2* *SERPINE1*	GDS3487
					*COL1A2*	*ADAMTS18*		
						*CST9*		
						*MMP10*		
						*SERPIND1*		
						*SERPINI1*		
FOXO1	LSK	Mouse	KD	*-*	*-*	*ADAMTS8*	*SERPINA7*	GDS2720
						*MMP12*	*CTSW*	
						*P4HTM*	*MMP12*	
						*SERPINA11*	*PRSS12*	
FOXO1	T reg	Mouse	KO	*-*	*COL9A3*	*ADAMTS6*	*ADAM8*	GSE40655
						*ADAMTS8*	*ADAM9*	
						*EGLN3*	*CST7*	
						*ITIH5*	*FAM20A*	
						*PAPPA2*	*PLG*	
							*SERPINB7*	
							*TGM2*	
FOXO1	CD8 T cells	Mouse	KO	*COL27A1*	*COL10A1*	*ADAMTS15*	*ADAMTS4*	GSE46025
						*CTSE*	*F10*	
						*SERPINA9*		
GATA1	Megakaryocytes	Mouse	KD	*-*	*-*	*ADAMTSL1*	*ADAM7*	GDS1245
						*F10*	*ADAMTS15*	
						*F13A1*	*EGLN2*	
						*MMP19*	*TGM2*	
						*SLPI*		
						*TGM3*		
GATA3	Basal breast cancer cells	Human	Ectopic expression	*COL8A1*	*COL4A5*	*CST2*	*CST6*	GDS4080
						*CST4*	*LOXL1*	
						*LOX*	*MMP1*	
						*PRSS12*	*MMP12*	
							*MMP3*	
							*PAPPA*	
							*PRSS3*	
							*TIMP4*	
GATA4	Endothelial-derived cells	Mouse	Inactivation	*-*	*-*	*ADAM9*	*CELA2A*	GDS3663
						*ADAMTS19*	*F10*	
						*F13B*	*PCSK6*	
						*HTRA4*	*SERPINA12*	
						*LEPRE1*	*SERPINB7*	
						*P4HA3*		
						*PLAU*		
						*SERPINA6*		
						*SULF2*		
						*TGM6*		
GATA4	Jejunum	Mouse	KO	*-*	*-*	*CD109*	*ADAM18*	GDS3486
						*LOXL3*	*ADAMTS7*	
						*SPAM1*	*LEPRE1*	
						*TMPRSS15*	*MEP1A*	
GATA4	Adult heart during pressure overload	Mouse	KO	*-*	*-*	*ADAM10*	*CTSK*	GDS4782
						*ADAM28*	*FAM20C*	
						*CTSC*	*ITIH5*	
						*CTSF*	*LEPREL1*	
						*MEP1A*	*PCSK6*	
						*MMP11*	*TGM1*	
						*MMP12*	*TGM2*	
						*MMP15*	*TIMP3*	
						*SERPINB8*	*TIMP4*	
JUN	B lymphoid cells	Mouse	KO	*-*	*-*	*ADAMTSL5*	*BMP1*	GDS4205
						*CTSE*	*OGFOD1*	
						*CTSO*	*SERPINB1*	
						*EGLN3*	*SERPINB5*	
						*LEPREL2*		
						*PLOD2*		
MYC	Medulloblastoma	Human	KD	*-*	*COL15A1*	*CTSL*	*CTSB*	GSE22139
					*COL1A1*		*HYAL4*	
					*COL4A1*		*ITIH5*	
					*COL5A1*		*LOX*	
					*COL5A3*		*MMP10*	
					*COL6A2*		*MMP7*	
							*MMP9*	
							*PLAU*	
							*SERPINE1*	
MYC	MCF10A cells	Human	OE	*-*	*-*	*CPN2*	*MMP7*	GSE43730
						*OGFOD1*	*PRSS12*	
						*SERPINB3*	*ST14*	
						*SERPINE1*		
						*SERPINE2*		
						*SULF1*		
NFE2L2	Liver	Mouse	KO	*COL15A1*	*COL22A1*	*CTSC*	*ADAMDEC1*	GDS514
				*COL2A1*		*EGLN2*	*CTSE*	
						*PRSS2*	*MMP9*	
						*SERPINF1*		
						*SERPINI1*		
STAT3	Cultured embryonic stem (ES) cells	Mouse	OE	*-*	*-*	*ADAM22*	*CTSG*	GDS3444
						*ADAM7*	*SERPINB1*	
						*MMP13*	*SERPIND1*	
						*MMP3*	*TLL1*	

## Conclusion

This review focused on the emerging evidence that longevity-signaling pathways promote extracellular matrix remodeling. There is a decline in ECM integrity during aging due to the accumulation of damage from collagen fragmentation, oxidation, or glycation. These changes in long-lived macromolecules of ECM can lead to the formation of cross-links and aggregates. A decline in ECM integrity leads to the loss of organ support and functions and can drive cellular aging and disease progression. We described in detail how ECM components influence the activity of some of the well-known longevity pathways, like mTOR, FOXO, Nrf2, and Sirtuins. Changes in ECM composition can cause dysregulation of longevity pathways and drive the progression of age-related disease. Moreover, we showed that many longevity transcription factors could regulate matrisome genes. Changes in the activity of longevity pathways can modify ECM composition, but the modification of ECM components can also change the activity of longevity pathways. In the future, it is necessary to develop new therapeutics that will directly target ECM components, possibly in combination with targeting longevity pathways to promote increased healthspan.
